# Thermal Evolution of Silicon and Fluorine Species in Fluorosilane-Modified Carbon Aerogel Composites

**DOI:** 10.3390/nano16191250

**Published:** 2026-10-03

**Authors:** Yiren Long, Junzong Feng, Yonggang Jiang, Yijie Hu, Jian Feng

**Affiliations:** 1 Science and Technology on Advanced Ceramic Fibers and Composites Laboratory, College of Aerospace Science and Engineering, National University of Defense Technology, Changsha 410073, China; 18779863283@163.com (Y.L.); jygemail@nudt.edu.cn (Y.J.); huyj2023@nudt.edu.cn (Y.H.);; 2 Hunan Provincial Key Laboratory of High-Speed Aerodynamic Metamaterials, Changsha 410073, China

**Keywords:** carbon aerogel composites, fluorosilane functionalization, high-temperature degradation, differential evolution of Si/F species, C_x_F_y_-containing volatile species, surface functional-layer failure

## Abstract

Carbon aerogels are widely used for thermal protection in extreme high-temperature environments under inert atmospheres because of their excellent thermal insulation. However, their high specific surface area and open-pore structure promote moisture uptake, necessitating hydrophobic or low-surface-energy functionalization for practical use. Existing studies have primarily focused on room-temperature wettability after modification, while the high-temperature decomposition of functional layers, elemental evolution, and volatile release remain insufficiently understood. Herein, vapor-phase fluorosilane (FAS)-modified carbon aerogel composites were investigated by combining solid-state chemical characterization with evolved-gas analysis to clarify the thermal evolution of the functional layer under inert high-temperature conditions. FAS modification produced a superhydrophobic surface with an initial water contact angle of approximately 163°. During heating, the FAS-derived functional layer entered a rapid-decomposition stage at approximately 372 °C, followed by the principal fluorinated-volatilization stage near 443 °C. Perfluoroalkyl-chain cleavage progressively removed fluorine from the material through the release of C_x_F_y_-containing volatile species, accompanied by the disappearance of C–F, CF_2_, and CF_3_ structures. In contrast, part of the silicon-containing fraction was retained and evolved into a Si–C-related residual structure. Although high-temperature exposure eliminated FAS-derived hydrophobicity, the nanoparticle-assembled nanoporous carbon framework remained intact without obvious structural collapse. These findings establish a selective thermal-failure pathway in which fluorinated chains preferentially decompose and volatilize, whereas silicon-containing species are partially retained and reconstructed on the carbon framework, clarifying the failure mechanism of surface-modified carbon aerogel nanomaterials in extreme thermal environments.

## 1. Introduction

Advanced aerospace vehicles, re-entry spacecraft, and deep-space exploration systems are exposed to sustained aerodynamic heating, steep thermal gradients, and prolonged thermal loads during service, requiring thermal protection materials to combine low weight with efficient insulation and high-temperature structural stability [1]. Aerogels, characterized by low density and nanoporous networks, are promising thermal insulators for extreme environments [2]. Among them, carbon aerogels combine low density with a nanoporous framework that suppresses solid- and gas-phase heat transfer and attenuates high-temperature infrared radiation. Their structural stability in inert atmospheres makes them attractive for thermal protection [3,4,5,6]. Practical service, however, also involves long-term storage, hygrothermal cycling, and complex interfacial environments. Because their open pores, high specific surface area, and surface-active sites are sensitive to water vapor and other external species [7,8,9], carbon aerogels often require functional surface layers that provide moisture resistance, contamination resistance, or interfacial regulation without compromising the insulating framework. Fluorosilanes combine low-surface-energy perfluoroalkyl chains with condensable silane end groups and are widely used to form moisture-resistant interfaces on porous materials [10,11]. Existing studies largely emphasize the wettability and environmental stability of these layers under ambient conditions, while their stability and failure pathways under extreme heating remain unclear. Critical questions include when fluorinated chains begin to decompose, how C–F bonds cleave, in which forms fluorine leaves the solid, and whether silicon volatilizes concurrently or forms a residual structure. The progression of these processes determines whether surface functionality fails before the supporting carbon framework loses structural integrity. Resolving the coupled degradation, elemental migration, and volatile release of fluorosilane-derived layers is therefore essential for assessing multifunctional carbon aerogels in extreme thermal environments.

Research on fluorinated siloxane functional layers has mainly addressed perfluoroalkylsilane films, fluorinated polymers, and functionalized porous materials. Layers formed by FOTS, FDTS, and PFOTS on SiO_2_, Si, and metal oxides generally comprise an interfacial Si–O–Si network and aligned CF_2_/CF_3_ segments. They retain siloxane connectivity and low surface energy at low to moderate temperatures, and moderate annealing can promote condensation and molecular ordering. With further heating, however, fluorocarbon chains become disordered, C–F-related signals and CF_2_/CF_3_ populations decline, and chain scission and segment removal lead to irreversible hydrophobicity loss [12,13,14,15,16,17,18,19,20,21]. This evolution depends on chain length, molecular packing, Si–O bonding, film defects, and atmosphere, reflecting distinct thermal responses of the fluorinated segments and siloxane network. Thermal-decomposition studies of PTFE, FEP, PFA, PVDF, and perfluorocarboxylic acids provide a mechanistic basis for fluorinated-chain volatilization. Main-chain C–C cleavage, end-group depolymerization, C–F activation, radical transfer, and β-scission generate CF_2_, CF_3_, and higher-carbon C_x_F_y_ intermediates. Subsequent disproportionation, recombination, cyclization, or reactions with oxygen and water can produce CF_4_, C_2_F_4_, C_2_F_6_, C_3_F_6_, other C_x_F_y_ species, HF, and COF_2_. TG–FTIR, TG–MS, and Py–GC–MS studies have found that inert environments generally favor low-molecular-weight perfluorocarbons, whereas oxygen or water promotes HF, COF_2_, CO, and CO_2_ formation. Precursor structure, temperature, residence time, particle size, and volatile mass transfer further affect product distributions [22,23,24,25,26,27,28,29,30,31,32,33,34,35]. In parallel, fluoropolymer thermolysis and thermal-processing studies have reported diverse fluorinated volatile species and persistent fluoroacids, while inhalation studies of PTFE pyrolysates have documented adverse pulmonary responses under defined exposure conditions [36,37,38]. These studies indirectly indicate that the fluorinated volatile species released during the thermal degradation of fluorosilane functional layers may pose potential environmental and health risks, highlighting the importance of identifying their evolution during high-temperature exposure. Collectively, these studies clarify possible bond-cleavage and radical-recombination pathways, but most concern planar films or bulk fluoropolymers. In thermal insulation materials, the functional layer is also influenced by the carbon skeleton, nanopore confinement, and interfacial reactions. Oxide-aerogel studies show that organic hydrophobic groups are removed first, followed by condensation and reconstruction of the residual siloxane network [39,40]. Carbon aerogels present a carbon/siloxane interface, open nanopores, and oxygen-containing sites that can alter volatile diffusion, residence time, and secondary reactions, potentially decoupling fluorine volatilization from silicon-residue reconstruction. Their high-temperature failure must therefore be examined by correlating solid-state evolution, gas-phase products, and elemental migration.

Herein, unmodified carbon aerogel composites (CCA) and vapor-phase FAS-modified carbon aerogel composites (FAS-CCA) were examined to clarify the failure of the low-surface-energy fluorinated siloxane layer in an inert high-temperature environment. Correlating solid-state chemical evolution with evolved-product release establishes the relationship among FAS-layer degradation, fluorine volatilization, and the evolution of silicon-containing species. The fluorocarbon chains undergo cleavage at intermediate temperatures, and fluorine leaves the solid mainly through C_x_F_y_-containing volatile species. By contrast, the silicon-containing fraction remains on the carbon skeleton and evolves into a Si–C-related residual structure, while the nanoparticle-based porous framework remains largely intact after treatment at 1500 °C. This work establishes a thermochemical pathway from fluorinated-chain degradation and fluorine volatilization to reconstruction of the silicon-containing residue, providing a mechanistic basis for the design and reliability assessment of surface-functionalized thermal insulation materials used in extreme environments.

## 2. Materials and Methods

### 2.1. Materials

Polyacrylonitrile (PAN)-based carbon fiber-reinforced carbon aerogel composites [41] were used as the substrates for vapor-phase hydrophobic modification. Before modification, the composites had an apparent density of 0.162 g·cm^−3^, a carbon fiber volume fraction of 3.4 vol%, and a specific surface area of 269.61 m^2^·g^−1^. The carbon aerogel composites were prepared according to our previous work [42]. 1H,1H,2H,2H-perfluorodecyltrimethoxysilane (FAS; ≥ 97%) was supplied by Shanghai Macklin Biochemical Co., Ltd. (Shanghai, China). FAS was used as the vapor-phase surface modifier.

### 2.2. Vapor-Phase Modification and Heat Treatment

Carbon aerogel composites (CCA), prepared according to our previously reported procedure, were dried at 120 °C for 1 d before vapor-phase modification. The FAS feed dosage was fixed at 15 wt% relative to the initial mass of CCA. The dried CCA and liquid FAS were placed separately in a sealed reactor to avoid direct contact. The sealed reactor was maintained at 120 °C for 2 d and then cooled to room temperature. The samples were subsequently dried at 120 °C for another 1 d to remove residual volatile precursor and weakly adsorbed species. After this post-drying treatment, the FAS-modified samples exhibited a net mass increase of 11.94 wt% relative to the initial CCA mass, indicating substantial retention of FAS-derived species within the porous framework. EDS analysis (Figure S1) further confirmed the incorporation of F and Si, with local contents of 16.08 wt% F and 1.17 wt% Si in the analyzed region. The unmodified and FAS-modified samples were denoted CCA and FAS-CCA, respectively.

For high-temperature treatment, FAS-CCA was heated to 1500 °C at 5 °C·min^−1^ under flowing N_2_, held at 1500 °C for 4 h in a tube furnace, and furnace-cooled to room temperature under N_2_. The resulting sample was denoted FAS-CCA-1500.

### 2.3. Characterization

Microstructures and framework morphologies before and after heat treatment were examined using a Sigma 300 scanning electron microscope (SEM; Carl Zeiss Microscopy GmbH, Oberkochen, Germany), and elemental distributions were mapped using the attached energy-dispersive X-ray spectrometer (EDS). Surface functional groups and chemical structures were analyzed using a Nicolet iS20 Fourier transform infrared spectrometer (FTIR; Thermo Fisher Scientific, Madison, WI, USA). Crystal structures and the structural evolution of the carbon and fluorinated components were characterized using a SmartLab SE X-ray diffractometer (XRD; Rigaku Corporation, Akishima, Japan). Surface elemental compositions and chemical states were analyzed using a K-Alpha X-ray photoelectron spectrometer (XPS; Thermo Fisher Scientific, East Grinstead, UK).

Evolved gases were analyzed using a TGA55+ thermogravimetric analyzer (TA Instruments, New Castle, DE, USA) coupled with a Fourier transform infrared spectrometer (Thermo Fisher Scientific, Madison, WI, USA). Samples were heated from 30 to 1000 °C at 10 °C·min^−1^ under N_2_ flowing at 50 mL·min^−1^. Mass changes, heat-flow behavior, and evolved-gas ion signals were recorded using a Discovery SDT 650 simultaneous DSC/TGA analyzer coupled with a Discovery mass spectrometer (SDT 650 + Discovery MS; TA Instruments, New Castle, DE, USA). TG–MS measurements were conducted from 30 to 1000 °C at 10 °C·min^−1^ under N_2_ flowing at 50 mL·min^−1^. Full-scan mass spectra and selected mass-to-charge ratio (*m*/*z*) signals were collected as functions of temperature to track the evolution of volatile products. Surface wettability was evaluated using an LSA100S-T contact angle/surface tension analyzer (LAUDA Scientific GmbH, Lauda-Königshofen, Germany) by measuring the water contact angle and rolling angle.

## 3. Results and Discussion

### 3.1. Structural Retention and Elemental Evolution After High-Temperature Treatment

**Figure 1:**
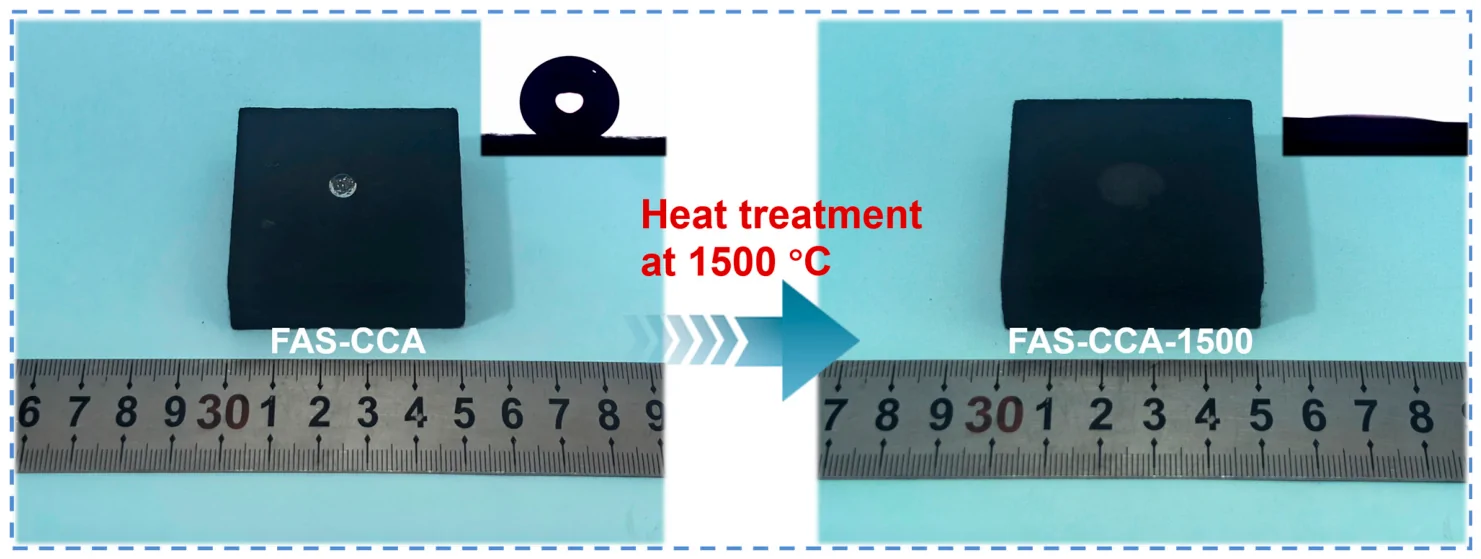
Macroscopic morphology and water-droplet behavior of FAS-CCA before and after heat treatment at 1500 °C under N_2_.

Figure 1 compares the macroscopic morphology and wetting behavior of the FAS-modified carbon aerogel composite before and after heat treatment at 1500 °C under N_2_. Before heat treatment, a water droplet retained a nearly spherical shape on FAS-CCA, with a water contact angle above 150°. This behavior confirms that vapor-phase modification introduced low-surface-energy fluorinated structures onto the pore-wall surfaces and strongly suppressed wetting of the porous framework. After heat treatment, the droplet spread and infiltrated the surface, reflecting the loss of FAS-derived hydrophobicity as the fluorinated organic chains decomposed and were removed. Despite this pronounced change in wettability, the overall dimensions and shape of the monolith remained nearly unchanged, with no obvious macroscopic shrinkage, warping, or structural damage. The N_2_ adsorption–desorption isotherms and pore-size distributions before and after heat treatment also remained highly similar (Figure S2), with no substantial change in the overall adsorption behavior or pore-size distribution, further confirming that the porous framework was largely preserved after exposure to 1500 °C. To further narrow the temperature range associated with the loss of hydrophobic functionality, additional wettability tests were performed after intermediate-temperature heat treatments (Figure S3). FAS-CCA retained hydrophobic behavior after treatment at 300 °C, whereas the water droplet spread rapidly over and penetrated into the surface after treatment at 400 °C, indicating that the hydrophobic function had already been lost at 400 °C. High-temperature exposure therefore primarily deactivated the surface functional layer rather than collapsing the carbon aerogel framework.

**Figure 2:**
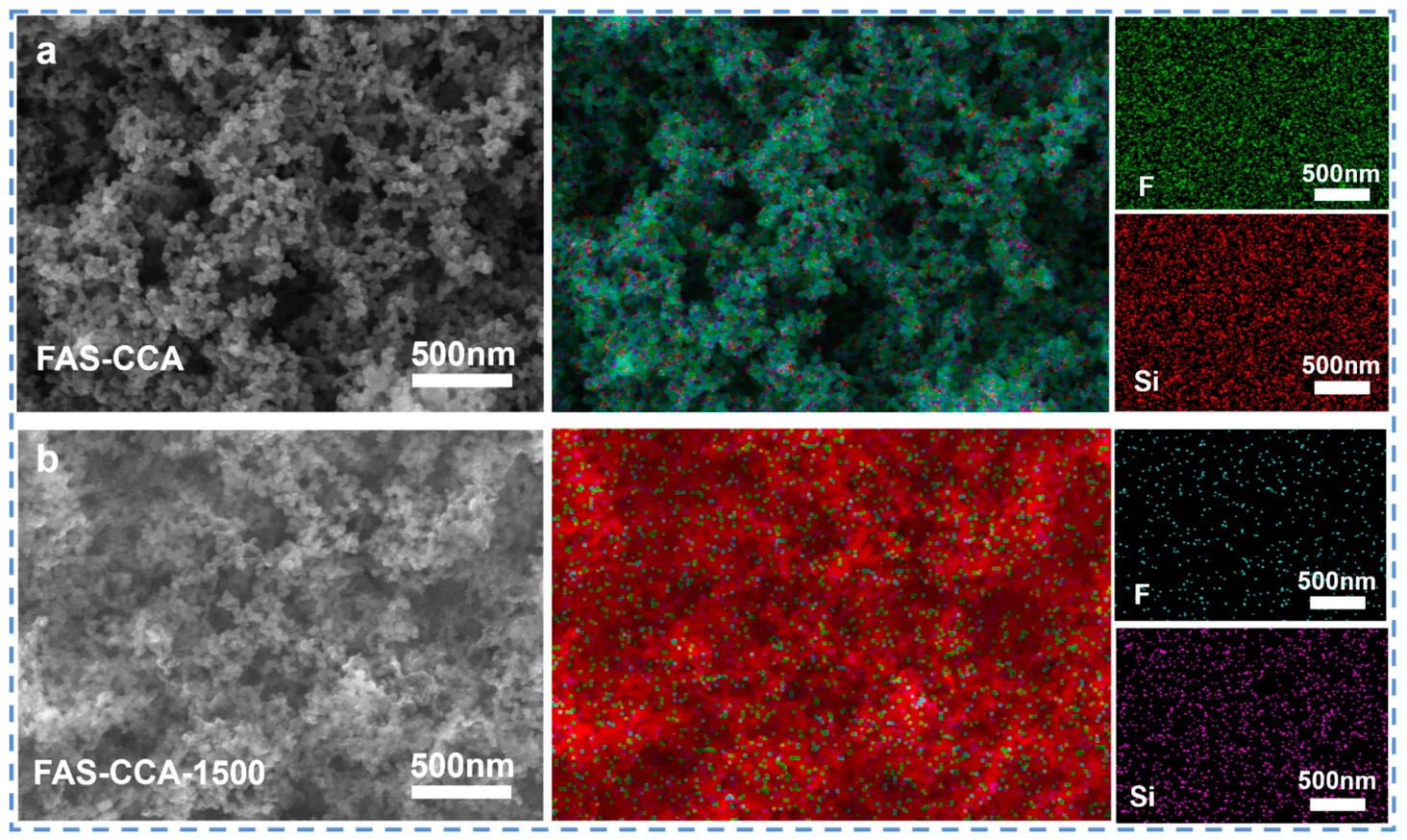
SEM images and EDS elemental maps of FAS-CCA before and after heat treatment at 1500 °C under N_2_: (**a**) FAS-CCA and (**b**) FAS-CCA-1500.

SEM and EDS analyses further resolved the associated microstructural and elemental changes. As shown in Figure 2a,b, FAS-CCA consisted of interconnected nanoparticles forming a continuous porous network. The particle framework and pore features remained clearly discernible after treatment at 1500 °C, without evident particle sintering, pore closure, or large-scale collapse. The loss of hydrophobicity was therefore governed by changes in surface chemistry rather than destruction of the nanoporous skeleton. Before heat treatment, both F and Si were distributed throughout the carbon aerogel framework, confirming the incorporation of FAS-derived species. After heat treatment, the F signal decreased markedly and only sparse features remained, whereas Si, although reduced, was still clearly detected within the carbon framework. The two elements thus followed distinct high-temperature pathways: the fluorinated organic component was preferentially removed from the solid, while part of the Si-containing component was retained. Although EDS does not resolve the chemical state of the residual Si, the contrasting F and Si signals establish their different retention behavior. Collectively, the macroscopic, SEM, and EDS results show that heat treatment eliminated the low-surface-energy function and depleted solid-phase F without compromising the nanoparticle-assembled porous framework while leaving a Si-containing residue on the carbon skeleton.

### 3.2. Chemical Evolution of Fas-Cca During High-Temperature Treatment

**Figure 3:**
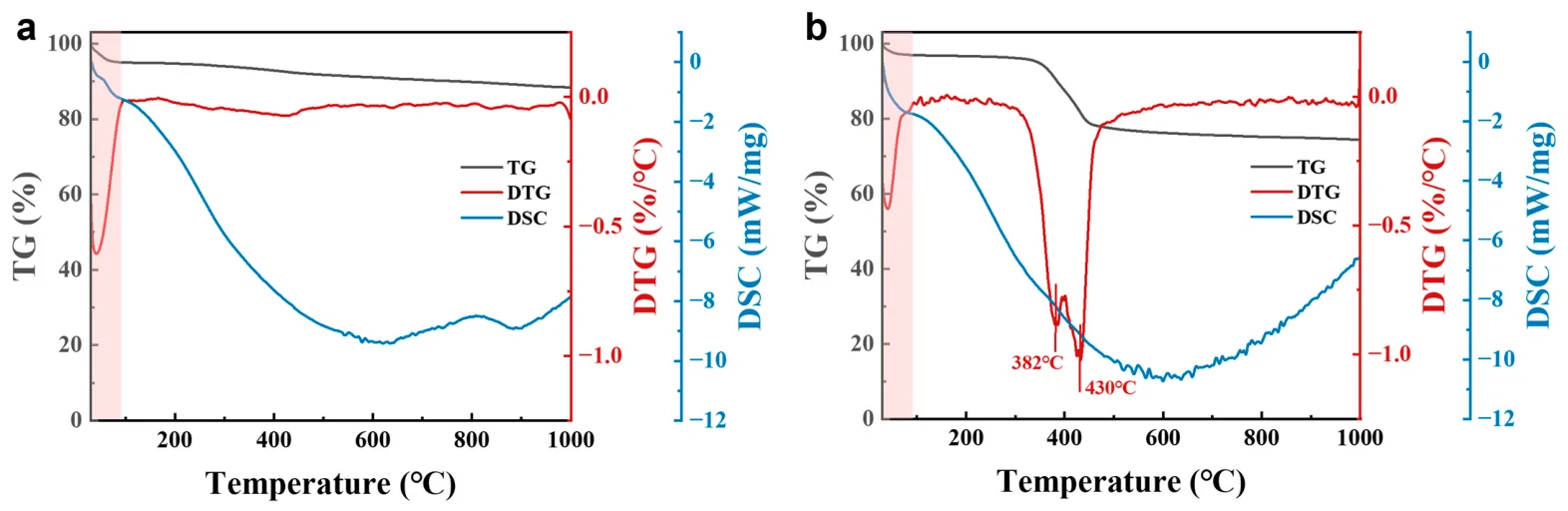
TG–DTG–DSC curves of CCA and FAS-CCA under N_2_: (**a**) CCA and (**b**) FAS-CCA.

The TG–DTG–DSC curves of CCA and FAS-CCA under N_2_ are presented in Figure 3a,b. CCA underwent only gradual mass loss from 30 to 1000 °C, with a small decrease at low temperature followed by slow variation upon further heating. The initial loss was mainly associated with adsorbed water and residual volatile species (highlighted by the red-shaded region in Figure 3), whereas the subsequent gradual decrease was attributed to the continued removal of oxygen-containing groups from the carbon surface. No pronounced DTG peak was observed, confirming the absence of a concentrated decomposition event and the high thermal stability of the carbon aerogel framework under N_2_. In contrast, FAS-CCA exhibited a distinct additional decomposition stage. Between 300 and 450 °C, CCA and FAS-CCA exhibited mass losses of 1.81 and 16.53 wt%, respectively, corresponding to an additional mass loss of approximately 14.72 wt% after FAS modification. EDS analysis (Figure S1) further showed that FAS modification introduced substantial amounts of F and Si into the material, with FAS-CCA containing 16.08 wt% F and 1.17 wt% Si before heat treatment. The combined F and Si content (17.25 wt%) is of the same order of magnitude as the additional TG mass loss of FAS-CCA, supporting that this extra mass loss is mainly associated with decomposition and volatilization of FAS-derived species. Thermal-stability studies of perfluoroalkylsilane films have shown that heating progressively decomposes and removes CF_2_ and CF_3_ structures from substrate surfaces [14,43]. Both DSC curves were dominated by broad endothermic responses without a distinct oxidation-related exotherm, consistent with desorption, bond cleavage, and volatilization rather than oxidative combustion of the carbon framework.

**Figure 4:**
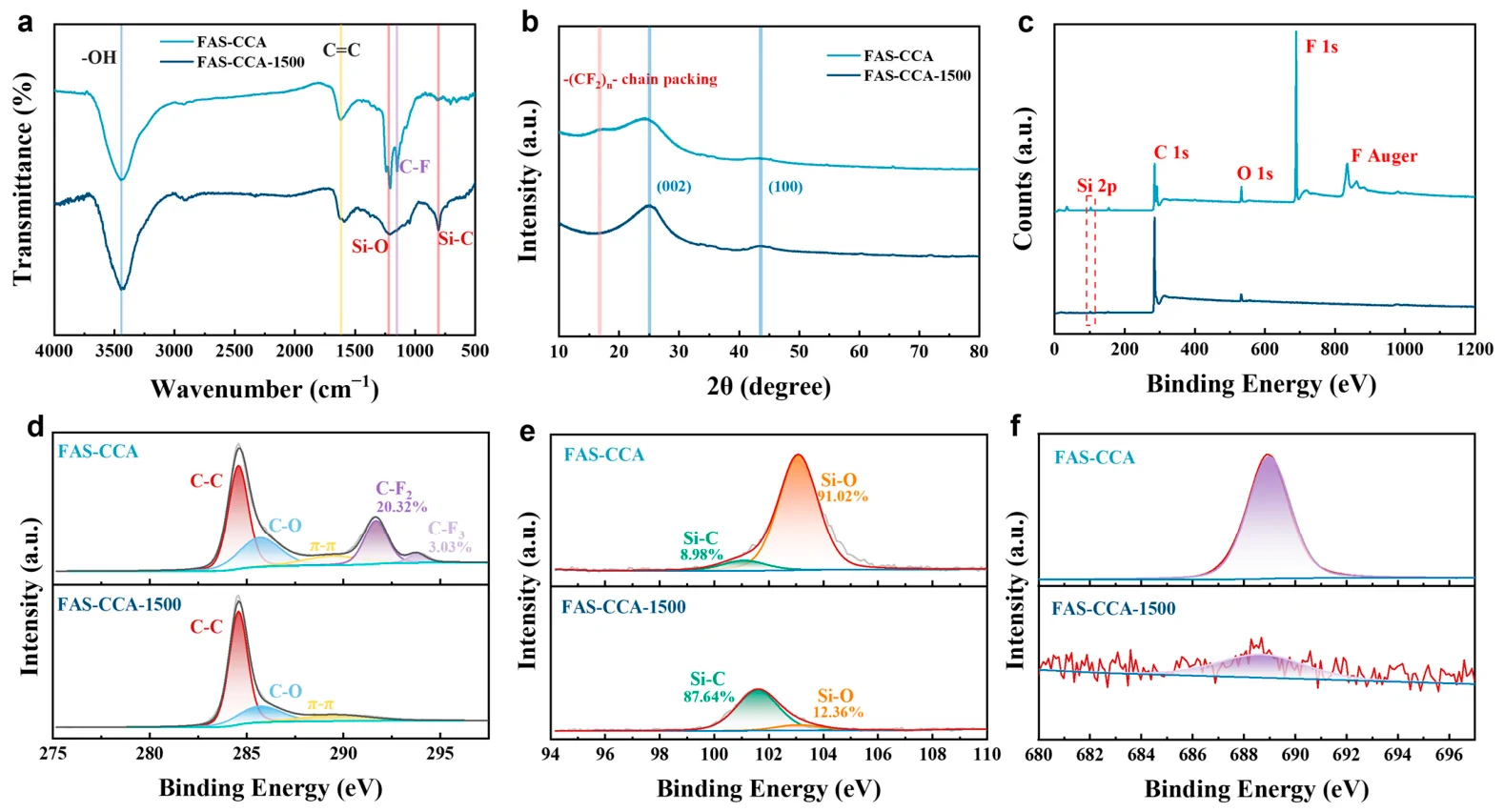
Chemical evolution of FAS-CCA before and after heat treatment at 1500 °C under N_2_: (**a**) FTIR spectra; (**b**) XRD patterns; (**c**) XPS survey spectra; and high-resolution (**d**) C 1s, (**e**) Si 2p, and (**f**) F 1s spectra.

The different thermal responses of the fluorinated chains and Si-containing component were further examined by FTIR (Figure 4a). Both FAS-CCA and FAS-CCA-1500 displayed a broad O–H stretching band near 3400 cm^−1^ and a carbon-framework C=C band near 1580 cm^−1^, confirming retention of the aromatic carbon network. In the fingerprint region of FAS-CCA, the bands at approximately 1200~1250, 1050~1100, and 800~850 cm^−1^ were assigned to C–F, Si–O-related, and Si–C-related vibrations, respectively. After treatment at 1500 °C, the C–F band disappeared, whereas the Si–O-related absorption weakened and the Si–C-related feature intensified. Thus, the fluorinated chains were cleaved and removed from the solid, while the residual Si-containing component evolved toward a Si–C-related local environment. The XRD patterns (Figure 4b) retained broad reflections at approximately 25° and 44°, corresponding to the (002) and (100) planes of disordered carbon, respectively [44]. Their continued dominance after heat treatment confirms that the framework remained amorphous or weakly graphitized. No distinct crystalline SiC reflection was detected; therefore, the available FTIR/XRD evidence supports a low-content or poorly ordered Si–C-related residual structure rather than bulk crystalline SiC. FAS-CCA also exhibited an additional feature near 17°. Long perfluoroalkyl chains possess high conformational rigidity and can develop partially ordered intermolecular packing [45]; this feature is therefore assigned to ordered –(CF_2_)_n_– chain packing within the FAS-derived layer. Its disappearance after treatment at 1500 °C confirms disruption of the perfluoroalkyl-chain structure.

XPS resolved the corresponding changes in elemental composition and bonding environment (Figure 4c–f). The survey spectrum of FAS-CCA contained C, O, Si, and F, including intense F 1s and F Auger signals. After heat treatment, the F-related signals vanished, whereas part of the Si signal remained. In the C 1s spectrum of FAS-CCA, CF_2_ and CF_3_ accounted for 20.32% and 3.03%, respectively. Their area ratio of approximately 6.7 closely matches the theoretical ratio of seven CF_2_ units to one terminal CF_3_ group in FAS, confirming the presence of intact perfluoroalkyl chains before heating. Both components disappeared after treatment, leaving a C 1s envelope dominated by C–C, C–O, and π–π* contributions. The Si 2p spectrum also changed markedly: the Si–O-related and Si–C-related fractions shifted from 91.02% and 8.98% in FAS-CCA to 12.36% and 87.64% in FAS-CCA-1500, respectively. Silicon oxycarbide systems can undergo Si–O/Si–C bond rearrangement and carbothermal conversion above 1400 °C under carbon-rich inert conditions [46]. The F 1s spectrum decreased from an intense peak to a signal close to the background. These solid-state analyses establish selective thermal evolution of the FAS-derived layer: fluorinated organic chains were preferentially removed, while part of the Si-containing component was retained as a Si–C-related residual structure without disrupting the carbon aerogel framework.

### 3.3. Gas-Phase Evolution During Thermal Decomposition of the Fas-Derived Layer

**Figure 5:**
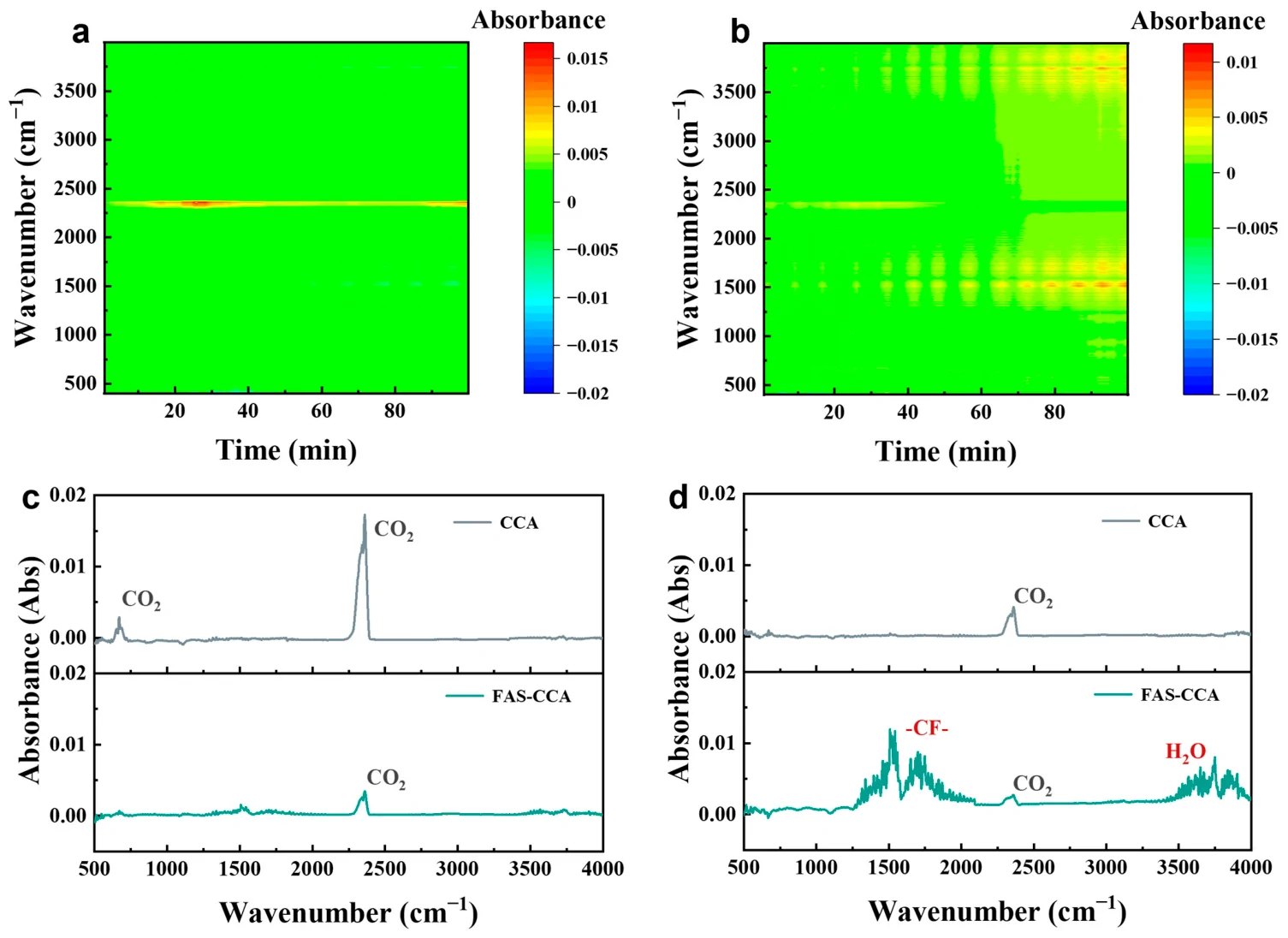
Gas-phase evolution of thermal decomposition products from CCA and FAS-CCA analyzed by TG–FTIR: two-dimensional absorption maps of (**a**) CCA and (**b**) FAS-CCA, and representative gas-phase FTIR spectra recorded at (**c**) 250 and (**d**) 800 °C.

TG–FTIR captured the dynamic release of volatile products during decomposition of the FAS-derived low-surface-energy layer. The two-dimensional spectrum of CCA (Figure 5a) was dominated by absorption between 2250 and 2500 cm^−1^, with the CO_2_ band near 2350 cm^−1^ reaching its maximum at approximately 250 °C. In conjunction with the gradual TG response, this low-temperature evolution is attributed to desorption and removal of residual oxygen-containing groups from the carbon surface. Under N_2_, carboxyl, anhydride, and less thermally stable carbonyl structures can release small amounts of CO_2_ through decarboxylation or carbon–oxygen rearrangement. In addition to CO_2_, FAS-CCA developed a markedly enhanced broad band across 1250–2000 cm^−1^, particularly within 1500~1750 cm^−1^ (Figure 5b). Although H_2_O bending near 1595 cm^−1^ contributes to this interval, the FAS-specific enhancement, together with the fluorocarbon fragment ions detected by TG–MS, supports a contribution from C_x_F_y_-containing volatile species generated during perfluoroalkyl-chain cleavage. This combined evidence identifies a fluorocarbon-related contribution. Strong H_2_O/O–H gas-phase absorption between 3500 and 4000 cm^−1^ was associated with the release of adsorbed water, dehydration of surface hydroxyl groups, and condensation of residual Si–OH species.

Representative spectra were extracted at 250 °C (Figure 5c), where CO_2_ evolution from CCA was strongest, and at 800 °C (Figure 5d), where FAS-CCA exhibited the most intense absorption within the region containing the proposed fluorocarbon-related contribution. At 250 °C, both CCA and FAS-CCA showed pronounced CO_2_ absorption, confirming that the low-temperature CO_2_ originated primarily from oxygen-containing structures in the carbon aerogel matrix. At 800 °C, FAS-CCA exhibited a strong broad band between 1250 and 2000 cm^−1^, accompanied by CO_2_ and H_2_O signals, whereas CCA retained only a weak CO_2_ response. The contrast shows that, after the rapid intermediate-temperature decomposition stage, FAS-derived fluorinated structures continued to undergo cleavage and gradual evolution of higher-molecular-weight fluorinated components.

**Figure 6:**
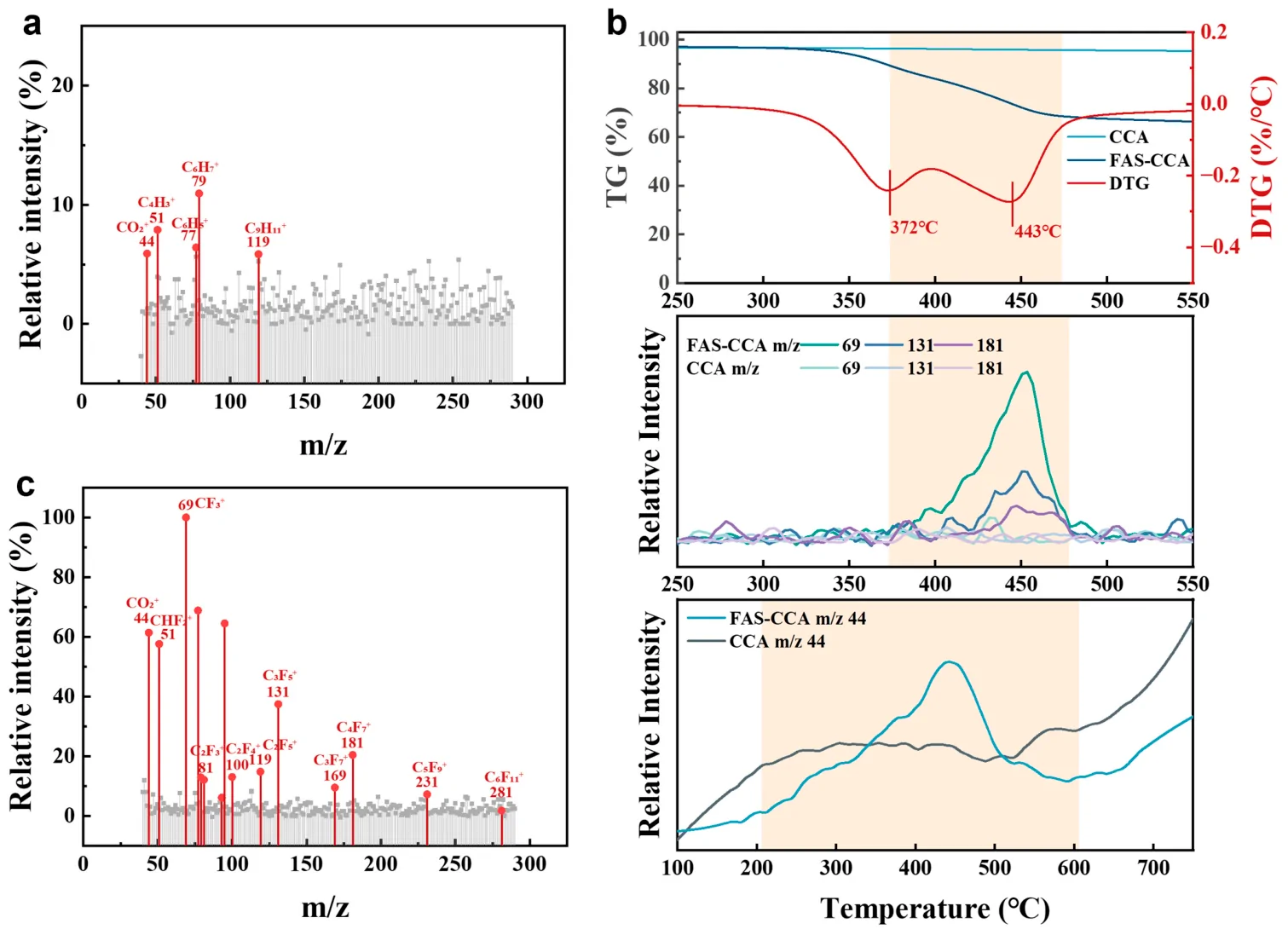
TG–MS analysis of volatile species released from CCA and FAS-CCA during thermal decomposition: (**a**) mass spectrum of CCA; (**b**) TG–DTG curves of FAS-CCA and temperature-dependent profiles of diagnostic fluorocarbon ions and *m*/*z* 44; and (**c**) mass spectrum of FAS-CCA.

TG–MS further provided fragment-ion evidence for the fluorinated origin of these gas-phase features. The mass spectrum of CCA (Figure 6a) was dominated by *m*/*z* 44 (CO_2_^+^), together with *m*/*z* 51 (C_4_H_3_^+^), 77 (C_6_H_5_^+^), 79 (C_6_H_7_^+^), and 119 (C_9_H_11_^+^), which are associated with neutral volatiles formed during removal of residual oxygen-containing groups and limited rearrangement of the carbonaceous structure. FAS-CCA exhibited a distinct series of fluorocarbon fragment ions (Figure 6c): *m*/*z* 51 (CHF_2_^+^), 69 (CF_3_^+^), 81 (C_2_F_3_^+^), 100 (C_2_F_4_^+^), 119 (C_2_F_5_^+^), 131 (C_3_F_5_^+^), 169 (C_3_F_7_^+^), 181 (C_4_F_7_^+^), 231 (C_5_F_9_^+^), and 281 (C_6_F_11_^+^). These assignments represent characteristic fragment ions generated during electron ionization rather than direct identification of the corresponding neutral molecules. Accordingly, the *m*/*z* 100 (C_2_F_4_^+^) signal is treated as a C_2_F_4_-related fragment rather than direct detection of neutral C_2_F_4_. The continuous distribution in carbon number and fluorination degree is consistent with C–C scission, chain-end depolymerization, and radical recombination of the long perfluoroalkyl chains, generating a mixture of C_x_F_y_-containing volatile species [47,48].

The temperature-dependent ion profiles further clarify the relationship between the TG–DTG mass-loss events and the evolution of fluorinated volatile species (Figure 6b). The first DTG peak appeared at approximately 372 °C, whereas the *m*/*z* 69 (CF_3_^+^ fragment), 131 (C_3_F_5_^+^ fragment), and 181 (C_4_F_7_^+^ fragment) signals began to increase at approximately 382 °C. Similarly, the second and more pronounced DTG peak occurred at approximately 443 °C, while the three fluorocarbon fragment-ion signals reached their maxima at approximately 453 °C. Thus, the MS response consistently lagged behind the corresponding TG–DTG features by approximately 10 °C. This systematic offset is attributed primarily to the transport and residence time of the evolved gases in the TG–MS transfer line, together with the response time of the mass spectrometer. Therefore, the TG–DTG and MS signals represent the same two-stage decomposition process of the FAS-derived fluorinated layer rather than separate thermal events (highlighted by the orange-shaded region in Figure 6b). The rise in the CF_3_^+^, C_3_F_5_^+^, and C_4_F_7_^+^ fragment signals during these two stages confirms the progressive cleavage of the perfluoroalkyl chains and the release of C_x_F_y_-containing volatile species. The *m*/*z* 44 profile followed a different trend: both samples released a low-temperature CO_2_ fraction at 200–300 °C, followed by a renewed increase above approximately 600 °C, consistent with TG–FTIR. Thus, CO_2_ primarily records the thermal evolution of the carbon framework and its oxygen-containing linkages, whereas FAS introduces a separate fluorocarbon volatilization pathway. Together, TG–FTIR and TG–MS support a dynamic sequence of initial chain scission, rapid release of C_x_F_y_-containing volatile species, and continued high-temperature evolution while accounting for the depletion of F from the solid FAS-derived layer.

## 4. Conclusions

This work systematically elucidates the failure mechanism of surface functionality in vapor-phase FAS-modified carbon aerogel composites under high-temperature conditions in N_2_. The low-surface-energy fluorinated siloxane layer formed by FAS on the carbon aerogel pore walls endowed the composite with superhydrophobicity (water contact angle, WCA ≈ 163°). After high-temperature treatment, this wetting-control function was largely lost, whereas the continuous porous carbon framework assembled from nanoparticles remained intact, without obvious sintering or structural collapse. The results show that the Si and F components of the FAS-derived low-surface-energy layer underwent pronounced differential evolution during heating. Upon heating, the functional layer entered rapid decomposition near 372 °C, and the principal release of fluorinated volatile species occurred around 443 °C. Progressive cleavage of the perfluoroalkyl chains eliminated the C–F, CF_2_, and CF_3_ structures, with fluorine leaving the material as C_x_F_y_-containing volatile species. Silicon did not volatilize synchronously; instead, part of the silicon-containing fraction remained on the carbon framework and evolved into a Si–C-related residual structure. Overall, this work clarifies the failure pathway of FAS-modified carbon aerogel composites under extreme thermal conditions and links the degradation of surface hydrophobicity with the distinct evolution of Si and F and the release of C_x_F_y_-containing volatile species. These findings provide a mechanistic reference for designing thermally stable functional surface layers, evaluating the service reliability of high-temperature insulation materials, and managing risks associated with fluorinated volatile emissions.

## Data Availability

The original contributions presented in this study are included in the article. Further inquiries can be directed to the corresponding author.

## Supplementary Materials

The following supporting information can be downloaded at: https://www.mdpi.com/article/10.3390/nano16191250/s1, Figure S1: EDS spectra of (a) FAS-CCA and (b) CCA; Table S1: EDS elemental composition of F and Si in FAS-CCA and CCA; Figure S2: (a) N_2_ adsorption–desorption isotherms and (b) pore-size distribution curves of CCA, FAS-CCA, and FAS-CCA-1500; Table S2: BET pore-structure parameters of CCA, FAS-CCA, and FAS-CCA-1500; Figure S3: Water contact angle images of FAS-CCA after heat treatment at (a) 300 °C. and (b) 400 °C.
