# Supplementary material for: Thermal Evolution of Silicon and Fluorine Species in Fluorosilane-Modified Carbon Aerogel Composites
Source: Nanomaterials (Basel). 2026 Oct 3;16(19):1250. doi: 10.3390/nano16191250 (PMC13667006; doi:10.3390/nano16191250)
Supplement: Supplementary file 1 [file nanomaterials-16-01250-s001.zip › nanomaterials-4603932-supplementary.pdf]

Article category: Research Article
Section: 2D and Carbon Nanomaterials

Thermal Evolution of Silicon and Fluorine Species in Fluorosilane-Modified Carbon Aerogel Composites

Yiren Long[ab], *Junzong Feng*[*ab], *Yonggang Jiang*[ab], *Yijie Hu*[ab], *Jian Feng*[ab]

[a] Science and Technology on Advanced Ceramic Fibers and Composites Laboratory, College of Aerospace Science and Engineering, National University of Defense Technology, Changsha 410073, P.R. China

[b] Hunan Provincial Key Laboratory of High-Speed Aerodynamic Metamaterials, Changsha, 410073, China

[*] Corresponding author.

E-mail: junzongfeng@nudt.edu.cn;

Supporting

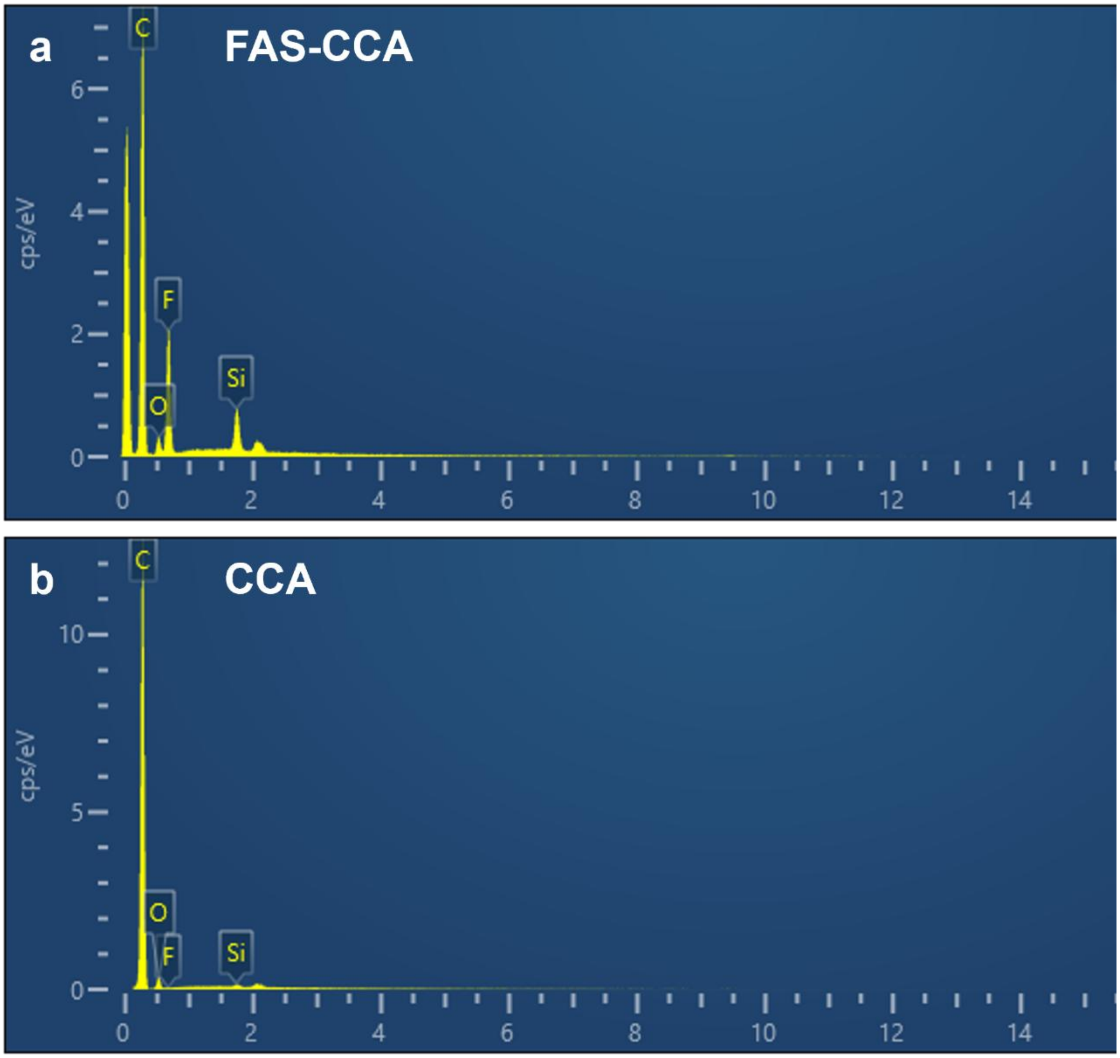

Figure.S1 EDS spectra of (a) FAS-CCA and (b) CCA.

Table.S1 EDS elemental composition of F and Si in FAS-CCA and CCA.

Sample	Element	Wt.%	At.%
FAS-CCA	F	16.08	11.01
	Si	1.17	0.54
CCA	F	0.01	0.01
	Si	0.11	0.05

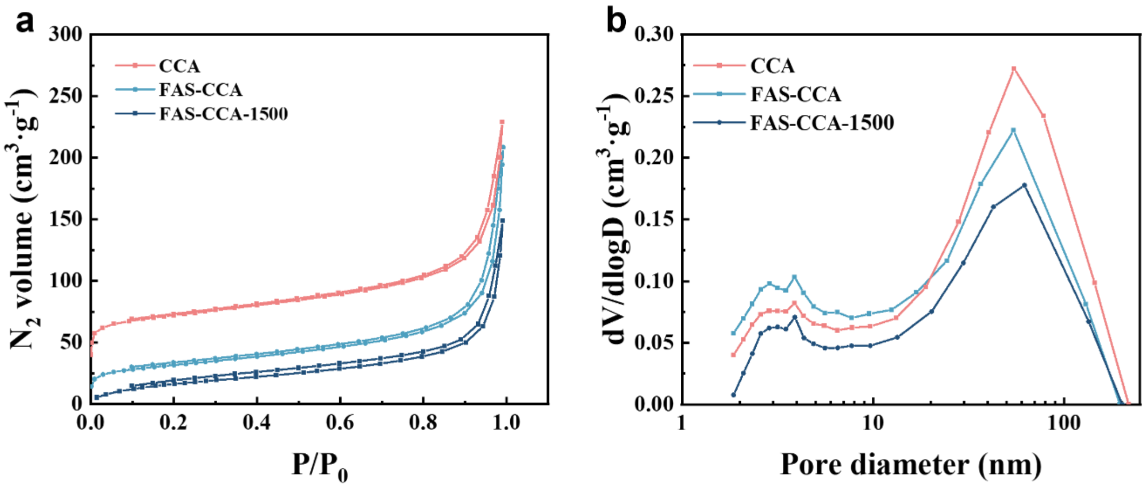

Figure.S2 (a) N_2 adsorption–desorption isotherms and (b) pore-size distribution curves of CCA, FAS-CCA and FAS-CCA-1500.

Table.S2 BET pore-structure parameters of CCA, FAS-CCA and FAS-CCA-1500.

Sample	S_{BET} (m^2/g)	S_{mes} (m^2/g)	S_{mic} (m^2/g)	V_{pore} (cm^3/g)	V_{mic} (cm^3/g)
CCA	269.61	95.25	174.36	0.3536	0.0700
FAS-CCA	113.35	80.91	32.45	0.3032	0.0137
FAS-CCA-1500	67.97	61.87	6.10	0.2139	0.0029

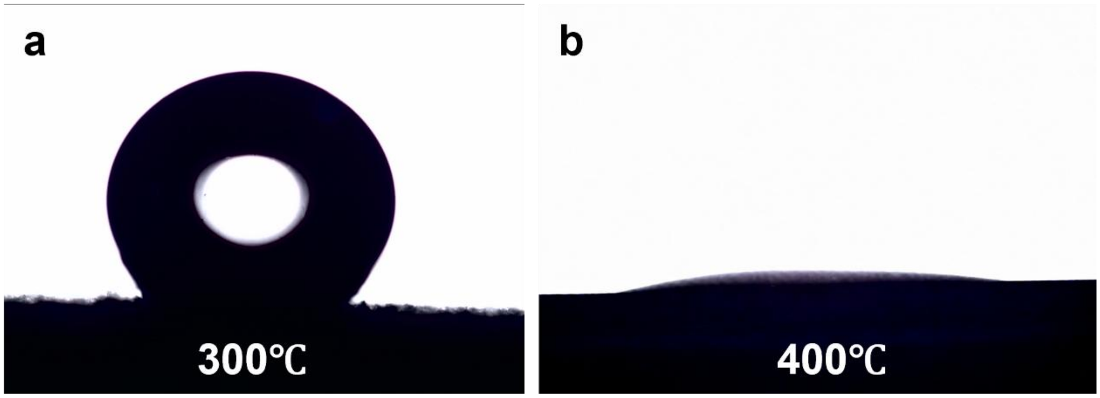

Figure.S3 Water contact angle images of FAS-CCA after heat treatment at (a) 300 °C and (b) 400 °C.
